# Influence of Histone Deacetylase Inhibitors and DNA-Methyltransferase Inhibitors on the NK Cell-Mediated Lysis of Pediatric B-Lineage Leukemia

**DOI:** 10.3389/fonc.2013.00099

**Published:** 2013-04-29

**Authors:** Matthias Manuel Pfeiffer, Helen Burow, Sabine Schleicher, Rupert Handgretinger, Peter Lang

**Affiliations:** ^1^Department of Pediatric Hematology and Oncology, University Children’s Hospital, Eberhard Karls University TuebingenTuebingen, Germany

**Keywords:** HDACi, DNMTi, NK cells, immunotherapy, pediatric lymphoblastic leukemia

## Abstract

Epigenetic drugs like histone deacetylase inhibitors (HDACi) and DNA-methyltransferase inhibitors (DNMTi) have been shown to be effective against a variety of tumor entities. Among different molecular anticancer activities of epigenetic active substances, up-regulation of natural killer (NK) cell ligands was described to contribute to an enhanced NK cell-mediated killing of tumor cell lines. So far, no data is available on this effect in childhood acute lymphoblastic leukemia. We investigated the effect of two HDACi [vorinostat, valproic acid (VPA)] and two DNMTi (azacytidine, decitabine) on the viability, expression of NK ligands, and NK susceptibility of the pre-B-cell-ALL cell line MHH-CALL-4. Whereas vorinostat, azacytidine, and decitabine directly reduced viability of the cell line, VPA had no direct cytotoxic effect. NKG2D-ligands were expressed only at very low levels and not affected by epigenetic treatment. Higher expression was found for the DNAM-1 ligands with significant up regulation of CD112 after treatment with VPA (*p* = 0.02). No significant increase in lysis mediated by resting NK cells could be observed, whereas incubation of target cells with decitabine resulted in a significant increase in lysis mediated by IL-2 activated NK cells (*p* = 0.0051, *p* = 0.06 for azacytidine). Vorinostat and VPA could increase the lysis by expanded NK cells which was statistically not significant due to high inter-individual variability. Furthermore, HDACi but not DNMTi reduced the NK-mediated lysis of MHH-CALL-4 after incubation of effector cells. In conclusion, there is a synergistic effect between epigenetic drugs and NK cells against MHH-CALL-4 which is not as strong as in other tumor entities. In situations where NK-mediated control of leukemia is assumed or wanted, a sophisticated combination of single epigenetic drugs and *ex vivo* expanded NK cells is needed to maximize the synergistic effect of both treatment strategies and DNMTIs may be preferred based on the direct inhibitory effect of HDACi on NK cell cytotoxicity.

## Introduction

Children with leukemia relapse after conventional chemotherapy still have a poor prognosis and will profit from stem cell transplantation (SCT). For patients without a family or matched unrelated donor haploidentical SCT from mismatched related donors has become an established procedure for the treatment of children with high risk and relapsed leukemia (Handgretinger et al., 2001; Lang et al., 2003; Marks et al., 2006). However, relapse after transplantation still represents a major problem. Natural killer (NK) cells are the lymphocyte subset showing the fastest reconstitution *in vivo*. Therefore, NK cells are the predominant lymphocyte subset which may exert antileukemic effects early after haploidentical SCT due to delayed reconstitution of a functional T cell repertoire. Indeed, NK cells have been shown to mediate antileukemic effects after haploidentical transplantation in adults with AML and children with ALL (Ruggeri et al., 2002; Leung et al., 2004). The function of NK cells is thereby regulated by the balance of activating and inhibitory signals transmitted by different cell surface receptors (Moretta et al., 2001; Lanier, 2005). One of the most important factors influencing NK-mediated lysis of pediatric ALL cells is the level of HLA class I molecules expressed by the leukemic cells (Pfeiffer et al., 2007). Strong HLA class I expression can engage inhibitory NK cell receptors which dampen signals transduced through activating receptors, whereas down regulation of HLA class I can render cells to valid targets for lysis by NK cells. Another way to overcome HLA class I mediated protection from lysis is the augmentation of activating signals. This can be achieved by activation of NK cells through cytokines which can lead to up-regulation of activating receptors like NGK2D or DNAM-1 or by up-regulation of ligands for activating NK receptors on leukemic cells. Epigenetic drugs like histone deacetylase inhibitors (HDACi) and DNA-methyltransferase inhibitors (DNMTi) have been shown to be effective against a variety of tumor entities. Among different molecular anticancer activities of epigenetic active substances an up-regulation of NK cell ligands was described for the HDACi valproic acid (VPA), suberoylanilide hydroxamic acid (SAHA, vorinostat), trichostatin A (TSA), and the DNMTi 5-aza-2′-deoxycytidine (decitabine), contributing to an enhanced NK cell-mediated killing of the different tumor entities (Rohner et al., 2007; Diermayr et al., 2008; López-Soto et al., 2009; Chávez-Blanco et al., 2011). Combination of activated and expanded NK cells with epigenetic drugs, which both have antitumor effects on their own, should result in a synergistic effect and a promising addition to conventional therapy and may enhance the NK-mediated anti leukemic effect after haploidentical transplantation. Therefore, we investigated the influence of the HDACi VPA and vorinostat and the DNMTi 5-azacytidine (Vidaza^®^) and 5-aza-2′-deoxycytidine (decitabine) on the cytotoxic function of NK cells, on the viability of the B-lineage acute lymphoblastic leukemia cell line MHH-CALL-4 and on the NK susceptibility of this cell line against resting and activated NK cells.

## Materials and Methods

### Cell lines

MHH-CALL-4 and K562 cells were obtained from the Leibniz Institute DSMZ-German Collection of Microorganisms and Cell Cultures (Braunschweig, Germany). K562mb15-41BBL cells were kindly provided by Dario Campana, St. Jude Children’s Research Hospital, Memphis, TN, USA). MHH-CALL-4 cells were cultured in RPMI 1640 supplemented with 20% FCS (both from Biochrom AG, Berlin, Germany), K562 were cultured in RPMI 1640 supplemented with 10% FCS and K562mb15-41BBL in RPMI 1640 supplemented with 10% human AB-serum (obtained from the Institute for Clinical and Experimental Transfusion Medicine, Tuebingen, Germany).

### HDACi and DNMTi

Vorinostat was kindly provided by MSD Sharp & Dohme GmbH, Haar, Germany. VPA was used from Desitin Arzneimittel GmbH (Hamburg, Germany). 5-Azacytidine and 5-aza-2′-deoxycytidine were obtained from Sigma Aldrich (Munich, Germany). HDACi and DNMTi were used in different concentrations, indicated in the different experiments. Target or effector cells were incubated for 48 h with HDACi or DNMTi before testing.

### Viability assay

The Cell Titer 96^®^ AQueous One Solution Cell Proliferation (MTS) Assay (Promega, Mannheim, Germany) was used to measure cell viability via redox enzyme activity, according to the protocol provided by the manufacturer. MHH-CALL-4 cells (100,000 cells/well) in the exponential growth phase were grown in 96-well plates. The day after seeding, the cells were incubated in the presence of HDACi or DNMTi for another 48 h at 37°C in a humidified atmosphere of 5% CO_2_ in air. At the end of the incubation period, MTS reagent (20 μl) was added to the wells, and the plate was incubated for 1 h protected from light. Absorbance was recorded at 490 nm using the Victor™ 1420 multilabel counter (Wallac, Rodgau, Germany). A reference wavelength of 630 nm was used to subtract background by excess cell debris or other non-specific absorbance. Wells containing the appropriate medium without cells served as blank.

### Flow cytometry

The following mAbs were used for flow cytometry: mouse IgG1 unconjugated, goat-anti-mouse-PE (Becton-Dickinson, Heidelberg, Germany), anti-MICA, anti-MICB unconjugated (kindly provided by Prof. Steinle, Institute for Molecular Medicine, Frankfurt am Main, Germany), anti-MICA/B APC, anti-CD112 PE (BioLegend, San Diego, CA, USA), anti-ULBP1 (Z-9), anti-ULBP2 (F16), anti-ULBP3 (2F9), anti-ULBP4 (6E6) (Santa Cruz Biotechnology, Dallas, TX, USA), anti-CD155 (eBioscience, San Diego, CA, USA), propidium iodide solution (Sigma Aldrich, Munich, Germany). Samples were analyzed on a FACSCalibur flow cytometer (Becton-Dickinson, Heidelberg, Germany) using CELLQUEST software (BD). A minimum of 20,000 events was used for assessment.

### Isolation and expansion of NK cells

Peripheral mononuclear cells (PMNC) from healthy donors were isolated by Ficoll–Hypaque density gradient centrifugation. Cells were enriched for CD56^+^ cells using CD56^+^ beads (Miltenyi Biotec, Bergisch–Gladbach, Germany) according to the manufacturer’s instructions. For expansion PMNC were incubated with irradiated (100 Gy) K562mb15-41BBL cells at a ratio of 1:1.5 in RPMI 1640 supplemented with 10% human AB-serum, l-glutamine, and 100 IU/ml interleukin-2 (Proleukin, Novartis, Basel, Suisse). Medium was exchanged every 2–3 days with fresh medium containing IL-2. Cells were cultured for 14 days.

### Cytotoxicity assay

Cytolytic activity of NK cells was tested in a 2-h BATDA [bis (acetoxymethyl) 2,2′:6′,2′-terpyridine-6,6″-dicarboxylate] europium release assay (Perkin Elmer, MA, USA). K562 and MHH-CALL-4 cells were used as target cells. Four different effector-to-target cell ratios were tested. Specific lysis was calculated as follows: % specific lysis = (experimental release − spontaneous release)/(maximum release − spontaneous release) × 100.

### Statistical analysis

Student’s *t*-tests were performed using GraphPad Prism version 4.01 for Windows, GraphPad Software, San Diego, CA, USA, www.graphpad.com.

## Results

### Influence of HDACi and DNMTi on viability of MHH-CALL-4

**Figure 1:**
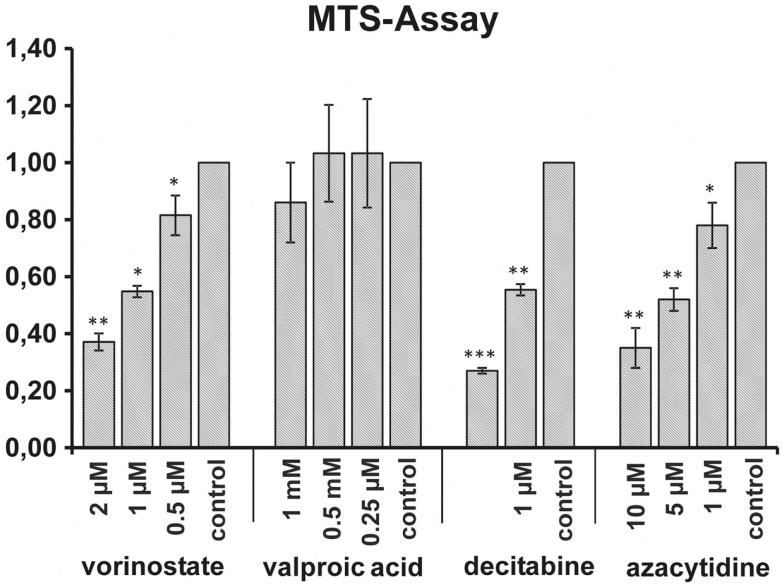
**Influence of HDACi and DNMTi on viability of MHH-CALL-4**. Incubation with vorinostat, decitabine, and azacytidine resulted in significant dose dependent reductions of cell viability whereas no reduction could be observed after incubation with valproic acid. Shown are mean values of absorbance ratio (absorbance of treated cells/absorbance of untreated cells) and standard deviation (*n* = 3 for vorinostat, *n* = 6 for valproic acid, *n* = 4 for decitabine and azacytidine, *p < 0.05, **p < 0.01, ***p < 0.005).

To test the direct effect of HDACi and DNMTi on the viability of the MHH-CALL-4 MTS assays were performed. Absorbance at 490 nm was measured after 1 and 3 h. Figure 1 shows the results of the measurement after 1 h. A significant reduction of viability could be observed after incubation with vorinostat (*t*-test, *p* = 0.002 for 2 μM), decitabine (*p* < 0.0001 for 2 μM), and azacytidine (*p* = 0.0031 for 10 μM) in a dose dependent manner. In contrary, VPA had no direct effect on the viability of the MHH-CALL-4 cells.

### Influence of HDACi and DNMTi on expression of NK ligands on MHH-CALL-4

**Figure 2:**
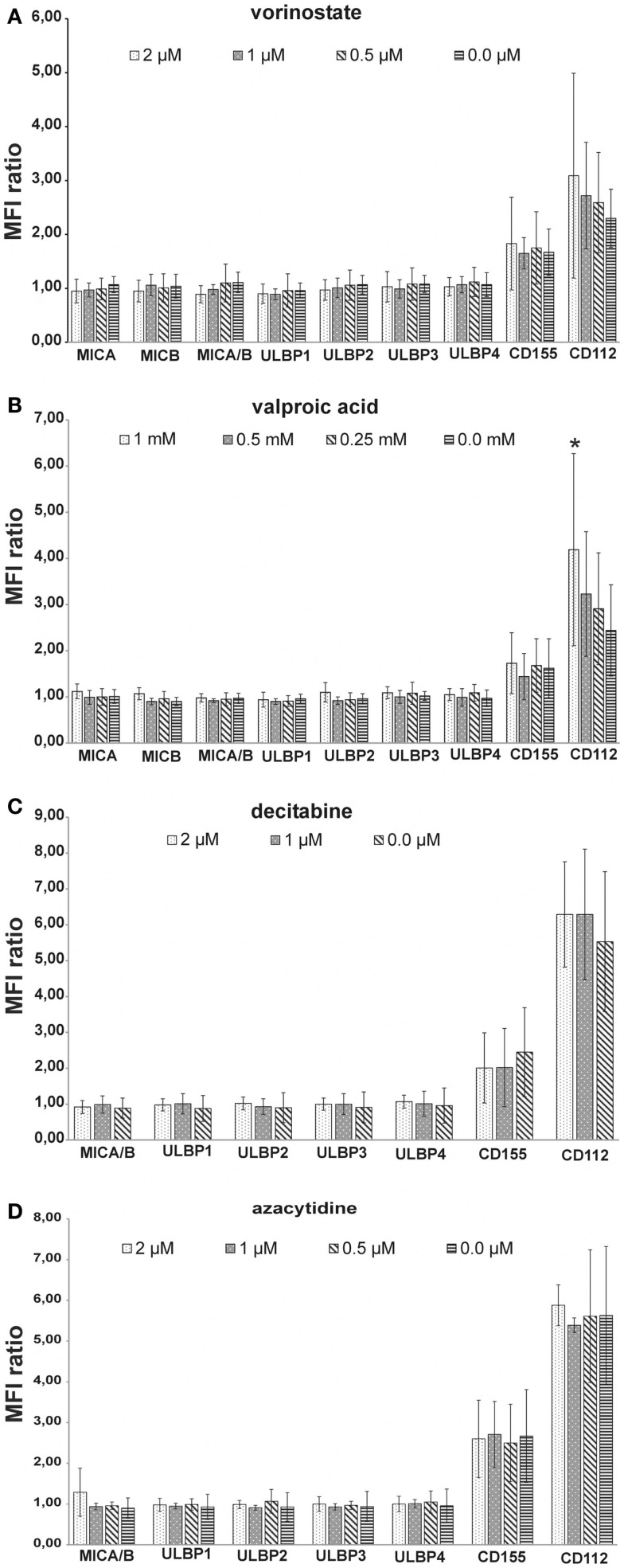
**Influence of HDACi and DNMTi on NK ligand expression of MHH-CALL-4**. Mean fluorescence intensity (MFI) was measured by FACS analysis and a MFI ratio (MFI with specific mAb/MFI with control mAb) was calculated. Shown are mean values and standard deviation [**(A)** vorinostate, *n* = 7; **(B)** valproic acid, *n* = 8; **(C)** decitabine, *n* = 4; **(D)** azacytidine, *n* = 4]. MHH-CALL-4 cells did not express ligands for NKG2D or only at very low levels. Higher expression was found for the DNAM-ligands CD112 and CD155 which were slightly up regulated by HDACi and not or only to a lower extend by DNMTi (*p < 0.05).

Histone deacetylase inhibitors have been described to up regulate the expression of ligands for activating NK receptors on different tumor entities. We analyzed the expression of the NKG2D-ligands MIC A, MIC B, ULBP1-4, and the DNAM-1 ligands CD112 and CD155 before and after incubation of MHH-CALL-4 cells with different concentrations of HDACi and DNMTi. Whereas the cells were negative or only low positive for the NKG2D-ligands, higher expression was found for the two DNAM-1 ligands CD112 and CD155 (Figure 2). Incubation with HDACi resulted in an increased expression of CD112, which reached a significant level only after incubation with VPA (*t*-test, *p* = 0.02 for 1 mM VPA, *p* = 0.22 for 2 μM vorinostat). DNMTi showed a different pattern without significant differences to the untreated control (*p* = 0.26 for 2 μM decitabine, *p* = 0.67 for 1 mM azacytidine). Expression of NKG2D-ligands on MHH-CALL-4 cells was not significantly changed by any of the tested substances.

### Influence of HDACi and DNMTi on NK susceptibility of MHH-CALL-4

**Figure 3:**
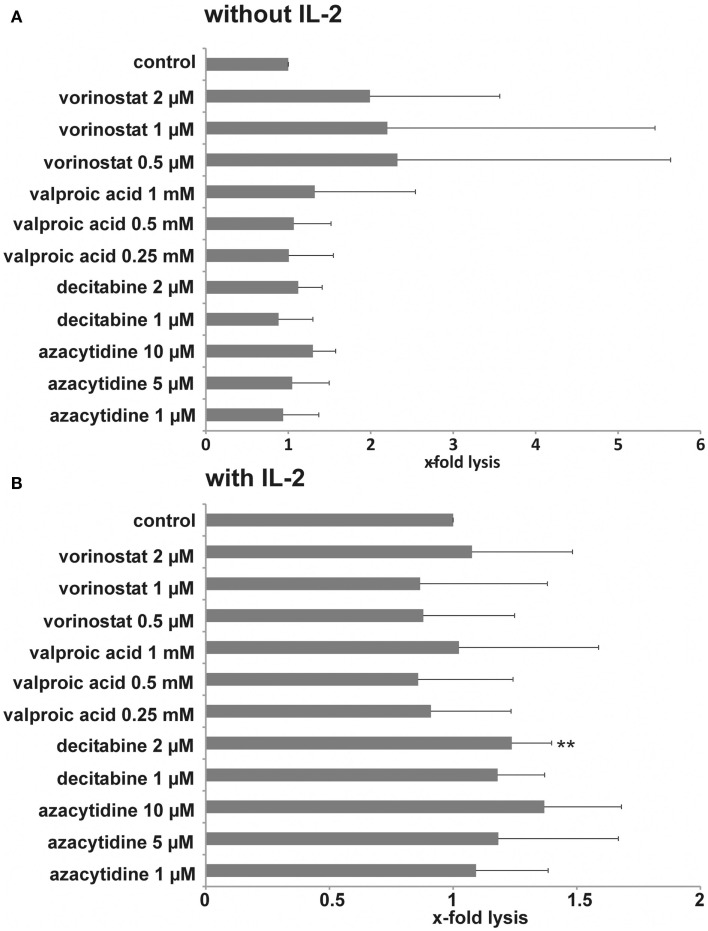
**Influence of HDACi and DNMTi on NK susceptibility of MHH-CALL-4 cells**. Leukemic cells were incubated with the indicated concentrations of HDACi and DNMTi for 48 h. Resting NK cells **(A)** or IL-2 stimulated NK cells **(B)** were used as effector cells. A lysis-ratio was calculated from each experiment as following: specific lysis with HDACi/DNMTi/specific lysis without HDACi/DNMTi. Shown are mean values and standard deviation from an effector-to-target cell ratio of 20:1 [*n* = 6 for vorinostat (four different donors), *n* = 15 for valproic acid (six different donors), *n* = 7 for decitabine (four different donors), *n* = 4 for azacytidine (four different donors), **p < 0.01].

**Figure 4:**
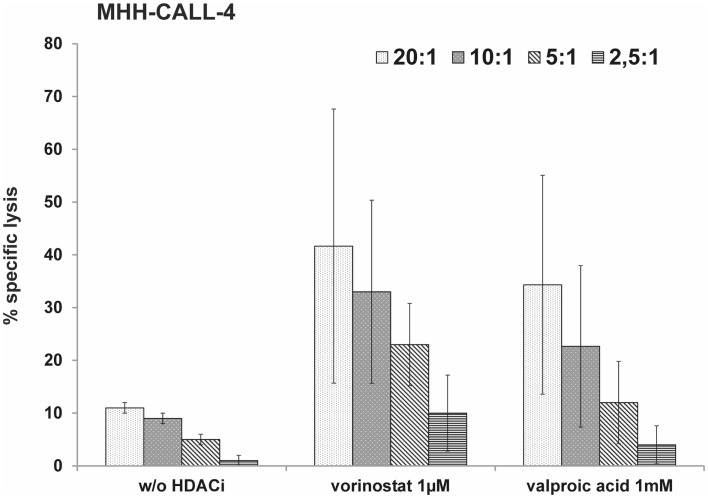
**HDACi can sensitize the MHH-CALL-4 cell line to lysis by expanded NK cells**. Leukemic cells were incubated with vorinostat or valproic acid for 48 h. Afterward, cytotoxicity assays were performed with *in vitro* expanded NK cells. Shown are mean values and standard deviation (*n* = 3).

Histone deacetylase inhibitors have been described to sensitize different tumor cell lines to a NK-mediated cell lysis by up-regulation of activating ligands. We tested cytotoxic activity of NK cells from healthy donors against pretreated MHH-CALL-4 cells. Incubation of the target cells with vorinostat resulted in the strongest increase in specific lysis by resting NK cells, which was statistically not significant due to a high variability between different donors and different experiments (*n* = 0.14 for 2 mM, *p* = 0.39 for 1 μM, *p* = 0.37 for 0.5 μM, Figure 3A). Incubation with VPA and DNMTi led to a smaller increase in specific lysis which also did not reach statistically significant levels (*p* = 0.65 for VPA 1 mM, *p* = 0.11 for decitabine 2 μM, *p* = 0.17 for azacytidine 10 μM). Stimulation with IL-2 led to a lower variability between different donors and lower standard deviation (Figure 3B). Statistically significant differences were observed after incubation with decitabine (*p* = 0.0051 for 2 μM, *p* = 0.08 for 1 μM). After incubation with azacytidine the increase was not statistically significant, which could be due to the smaller number of experiments (*p* = 0.06 for 10 μM). Finally, *in vitro* expanded NK cells were used against target cells pretreated with HDACi (Figure 4). A clear increase in cell lysis after incubation with HDACi could be observed with these effector cells but was not statistically significant due to the small number of experiments and high variability between different donors.

### Influence of HDACi and DNMTi on NK cell activity

**Figure 5:**
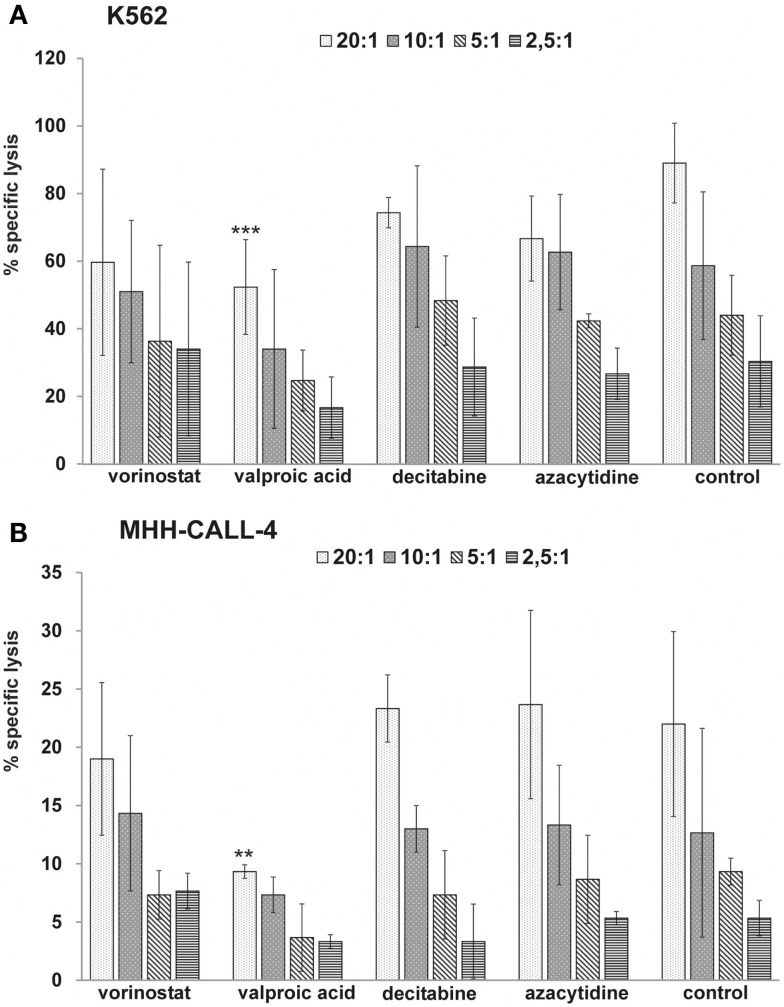
**HDACi but not DNMTi reduce the NK-mediated lysis of K562 and MHH-CALL-4 cells**. Shown are mean values and standard deviation from three independent assays with treated NK cells from healthy donors against untreated K562 **(A)** and untreated MHH-CALL-4 **(B)** (**p < 0.01, ***p < 0.005).

To test the direct effect of HDACi and DNMTi on cytotoxic function of NK cells, effector cells were pretreated with HDACi and DNMTi and tested in cytotoxicity assays against untreated K562 and MHH-CALL-4 (Figure 5). Both HDACi reduced the cytotoxic capacity of the NK cells against K562 and MHH-CALL-4 with a stronger and significant reduction after incubation with VPA (E:T = 20:1, *p* = 0.0002 for K562, *p* = 0.008 for MHH-CALL-4). DNMTi did not significantly alter NK activity against both K562 and MHH-CALL-4 cells. Furthermore, incubation with HDACi reduced the expression of NKG2D, NKp30, and NKp46, while incubation with DNMTi did not affect the expression of these receptors (data not shown).

## Discussion

Histone deacetylase inhibitors and DNMTi showed a direct cytotoxic effect on MHH-CALL-4 cells with exception of VPA. In other studies, VPA also showed a direct effect against different human leukemia cell lines (Kawagoe et al., 2002; Sakajiri et al., 2005). Taken together, there is concordant evidence that HDACi are capable of inducing apoptosis not only in AML but also in T- and B-cell-precursor-cell lines, providing a strong rationale for evaluation of these substances in preclinical models of ALL. Vorinostat and VPA also showed reduction of leukemic cell growth in a NOD/SCID mouse model of childhood acute lymphoblastic leukemia (Einsiedel et al., 2006). Furthermore, synergistic effects of HDACi and DNMTi with conventional chemotherapy have been shown and a combined pretreatment with vorinostat and decitabine resulted in even greater cytotoxicity of chemotherapy compared to each agent alone (Yang et al., 2005; Leclerc et al., 2010; Bhatla et al., 2012). Data from adult trials show that HDACi, in monotherapy as well as in combination therapy, are generally well tolerated and similar results were obtained in a phase I study in children and adolescents with solid tumors or leukemia (Fouladi et al., 2010). Therefore, incorporation of these epigenetic agents to the standard chemotherapy could be a promising approach to the treatment of, e.g., relapsed pediatric acute lymphoblastic leukemia.

Most pediatric patients with very high risk leukemia or early relapse after conventional chemotherapy will receive SCT. In several studies it was shown that NK-mediated leukemia control plays an important role after autologous and allogeneic transplantation (Lowdell et al., 2002; Ruggeri et al., 2002; Leung et al., 2004). Furthermore, reconstitution pattern of NK cell receptors and NK-mediated cytotoxic activity were correlated to relapse rate after haploidentical SCT in children (Pfeiffer et al., 2010; Lang et al., 2011). Studies with several solid tumor entities and AML have shown that treatment with HDACi and DNMTi could up regulate the expression of activating NK cell ligands, contributing to an enhanced NK cell-mediated killing of the different tumor entities (Rohner et al., 2007; Diermayr et al., 2008; López-Soto et al., 2009; Chávez-Blanco et al., 2011). Here, we showed that this effect is not as pronounced in MHH-CALL-4 cells. MHH-CALL-4 cells were either negative or only very low positive for the different NKG2D-ligands. In contrast, we found higher expression of the DNAM-1 ligands CD112 and CD155. This is in line with findings from Pende et al. (2005) which obtained comparable results on ALL blast from different patients and own unpublished results, where leukemic blasts from 21 patients with precursor B-cell-acute lymphoblastic leukemia were analyzed. NKG2D-ligands could not be up regulated through incubation with vorinostat, VPA, azacytidine, or decitabine. The expression level of the DNAM-1 ligand CD112 could be further elevated by incubation with HDACi. The leukemic cells could not be significantly sensitized to the lysis by resting NK cells which can be due to high inter-individual variability of different NK cell donors. Using IL-2 stimulated NK cells we found a statistically significant effect for decitabine and a nearly statistically significant effect for azacytidine (*p* = 0.06). A clear but also not statistically significant effect was observed with HDACi when using *in vitro* expanded NK cells as effectors. A limitation of our study is the use of only one ALL cell line. Unfortunately, primary blasts from our patients were not stable enough in culture to investigate an effect of HDACi or DNMTi over 48 h.

Beside the effect on target cells, epigenetic drugs can also affect the effector cells. In our experiments HDACi reduced the NK-mediated lysis of both the standard NK target K562 and MHH-CALL-4 cells and reduced the expression level of activating NK receptors on the cell surface. Comparable results against K562 and other cell lines have been previously reported (Ogbomo et al., 2007; Rossi et al., 2012). In contrast, DNMTi did not significantly influence the NK-mediated lysis of K562 and MHH-CALL-4 in our experiments. Different results have been published by Schmiedel et al. (2011) showing a reduction of NK-mediated lysis after incubation with azacytidine and an increase after incubation with decitabine. The different observations could be due to different incubation periods (24 vs. 48 h), different NK cell preparation (expanded NK cells with IL-2 and RPMI 8866 feeder cells vs. freshly isolated NK cells in our experiments). Also – as in our experiments – there was great variability between different NK donors and mean lysis of K562 without treatment was clearly higher in the azacytidine experiments compared to the decitabine experiments and hence maybe contributed to the different effect. On the other hand, the observed effect was also shown for cytokine production. Recently published data from Kopp et al. (2013) showed a significant inhibition of NK-mediated cytotoxicity by decitabine at intermediate concentrations (0.1–2.5 μM) with a *U*-shaped dose response curve and only little effects at low or high concentrations of decitabine. These findings could also explain the different results obtained by different groups and suggest further investigation *in vivo* to determine optimal dosages of decitabine.

The diverse effects of the different epigenetic drugs on NK activity as well as the diverse effects of the same drugs with different NK cell donors complicate recommendations for a clinical use. Especially in clinical situations where NK-mediated leukemia control is assumed or wanted – like after haploidentical transplantation – a sophisticated combination of single epigenetic drugs and, e.g., *ex vivo* expanded NK cells is needed to maximize the synergistic effect of both treatment strategies and should be based on individual testing. In this context, DNMTIs may be preferred due to the stronger inhibitory effect of HDACi on NK cell cytotoxicity.

## Conflict of Interest Statement

MMP had a research grant from MSD Germany supplying vorinostat and financial support for the conducted experiments. All other authors have no financial or commercial and personal interest or relationships to disclose with other people or organizations that could inappropriately influence or bias their work.
